# Water Deficit Improves Reproductive Fitness in *Nicotiana benthamiana* Plants Infected by *Cucumber mosaic virus*

**DOI:** 10.3390/plants11091240

**Published:** 2022-05-04

**Authors:** Marina Moreno, Belén Ojeda, Francisco J. Hernández-Walias, Eugenio Sanz-García, Tomás Canto, Francisco Tenllado

**Affiliations:** Departamento de Biotecnología Microbiana y de Plantas, Centro de Investigaciones Biológicas Margarita Salas, CSIC, 28040 Madrid, Spain; marinamoreno55@gmail.com (M.M.); b.ojeda@edu.uah.es (B.O.); hwfcojavier32@hotmail.com (F.J.H.-W.); eugeniosanz97@gmail.com (E.S.-G.); tomas.canto@cib.csic.es (T.C.)

**Keywords:** water deficit, virus infection, combined abiotic and biotic stresses, reproductive fitness, tolerance to drought, tolerance to virus, climate changes

## Abstract

Plants are concurrently exposed to biotic and abiotic stresses, including infection by viruses and drought. Combined stresses result in plant responses that are different from those observed for each individual stress. We investigated compensatory effects induced by virus infection on the fitness of hosts grown under water deficit, and the hypothesis that water deficit improves tolerance, estimated as reproductive fitness, to virus infection. Our results show that infection by *Turnip mosaic virus* (TuMV) or *Cucumber mosaic virus* (CMV) promotes drought tolerance in *Arabidopsis thaliana* and *Nicotiana benthamiana*. However, neither CMV nor TuMV had a positive impact on host reproductive fitness following withdrawal of water, as determined by measuring the number of individuals producing seeds, seed grains, and seed germination rates. Importantly, infection by CMV but not by TuMV improved the reproductive fitness of *N. benthamiana* plants when exposed to drought compared to watered, virus-infected plants. However, no such conditional phenotype was found in Arabidopsis plants infected with CMV. Water deficit did not affect the capacity of infected plants to transmit CMV through seeds. These findings highlight a conditional improvement in biological efficacy of *N. benthamiana* plants infected with CMV under water deficit, and lead to the prediction that plants can exhibit increased tolerance to specific viruses under some of the projected climate change scenarios.

## 1. Introduction

Plants are exposed to a diverse range of biotic and abiotic stresses that do not occur separately in time but are commonly present simultaneously [1,2]. The outcomes of multiple stresses can provide either tolerance or increased susceptibility to any of the stresses depending on the plant species, developmental stage and stress severity [3,4]. Although plant viruses give rise to many important diseases in crops worldwide, losses in agriculture due to abiotic stresses such as cold, salinity, heat and drought generally exceed those caused by viruses [5]. In particular, stress caused by water deficit is one of the major threats that affect plant physiology and growth, particularly owing to the increase of drought episodes caused by global warming [6]. Plants have developed a range of approaches to buffer the negative impact of drought on their physiology [7]. At an early stage of water stress, water content is kept within relatively narrow limits by increasing water capture and by limiting water loss from evapotranspiration by partially closing stomata. Stomatal closure reduces the entry of CO_2_, consequently decreasing photosynthesis and productive capacity. The effect of water deficit on plants leads to profound changes in hormones and secondary metabolites involved in plant defenses [8,9], the outcomes of which for plant resistance to pathogens are largely unexplored.

Several lines of evidence show that certain combinations of biotic and abiotic stresses could confer a positive effect on plant performance by increasing the tolerance to abiotic stresses. In particular, mechanisms and processes operating in compatible plants–virus interactions between might offer plants a better performance under abiotic stresses. For instance, it has been reported that infection by viruses can enhance the tolerance of host plants to cold and drought [10,11,12,13,14,15]. In addition, infection of Arabidopsis by different viruses rendered seeds with improved tolerance to deterioration by elevated temperature [16]. The reason for this increased tolerance to abiotic stresses in plants infected with viruses might reside in that plants made use of interlinked signaling pathways to respond to different environmental stresses, and that several of these responses are common in tolerance against virus and abiotic stresses [8,17].

On the other hand, abiotic stresses (some of them associated with global warming, i.e., elevated O_3_, CO_2_ and temperature, drought) can modulate plant tolerance toward pathogens by mechanisms that include changes in the response of plants to changing environmental conditions [18,19]. For instance, a lower incidence of virus diseases and severity of symptoms was accompanied by modulated defenses in plants grown at elevated CO_2_ [20,21,22]. Bilgin et al. [23] reported that ozone stress enhanced soybean tolerance to *Soybean mosaic virus*. Elevated temperatures have been described to cause a weakening and even a masking of symptoms in some compatible plant–virus interactions [24]. With regard to drought, it has been reported that the dynamics of symptoms and virus spread can be altered by water deficit in Arabidopsis infected with *Cauliflower mosaic virus* (CaMV) [25,26]. Indeed, survival of plants infected by CaMV grown under water deficit was increased when compared to plants grown under normal irrigation. However, the vegetative performance of CaMV-infected plants under water deficit could not be correlated with a better performance during the reproductive phase, since none of the infected plants developed seeds. Heat and drought tolerance mechanisms mediated by an increase in osmolytes may be common themes in plant tolerance to heat and drought [1].

The preferential survival of virus-infected plants under water deficit represents an advantage in biological efficacy (fitness) only if the survivors can subsequently produce offspring at higher rates than infected plants grown under watered conditions [12,27]. In this sense, it has been reported that virus-induced tolerance to drought was not always correlated with an increase in fecundity, with virulence being detrimental to reproductive fitness (defined as the relative success of an individual to pass on its genes to the subsequent generations) [10,28]. In addition, enhanced survival of the infected plants under water deficit could represent an advantage for the virus through increased opportunities to spread in the plant population, either by an increased duration of the vegetative phase or via propagation by seeds [26]. In this study, we utilized *Cucumber mosaic virus* (CMV) and *Turnip mosaic virus* (TuMV), and *Arabidopsis thaliana* and *Nicotiana benthamiana*. Both viruses are commonly found in wild populations of Arabidopsis, indicating that the Arabidopsis–TuMV and Arabidopsis–CMV pathosystems are significant in nature [29]. *N. benthamiana* has been adopted as a model plant by virologists due to its general susceptibility to virus infection. It is an allotetraploid species from the Suaveolentes section, resulting from the hybridization of a maternal progenitor of section Noctiflora, and a member of section Sylvestres as a paternal subgenome donor [30]. Transmission of CMV through seeds in *N. benthamiana* and Arabidopsis and of TuMV in Arabidopsis has been well established [31], but no data have been reported on seed transmission of TuMV in *N. benthamiana*. The aim of this work was to investigate compensatory effects induced by virus infection on host fitness when grown under water deficit, and to test the hypothesis that water deficit improves tolerance, estimated as reproductive fitness, to virus infection. In addition, we also analyzed the effect of water deficit on virus transmission through seeds. Our results show that infection by TuMV or CMV promoted drought tolerance in Arabidopsis and *N. benthamiana*. More importantly, infection by CMV but not by TuMV improved the reproductive fitness of *N. benthamiana* when exposed to drought compared to watered, virus-infected plants. However, no such conditional phenotype was observed in Arabidopsis plants infected with CMV. Water deficit did not affect seed transmission of CMV in *N. benthamiana* and Arabidopsis plants.

## 2. Results

### 2.1. Tolerance to Drought in Virus-Infected N. Benthamiana Plants

**Figure 1:**
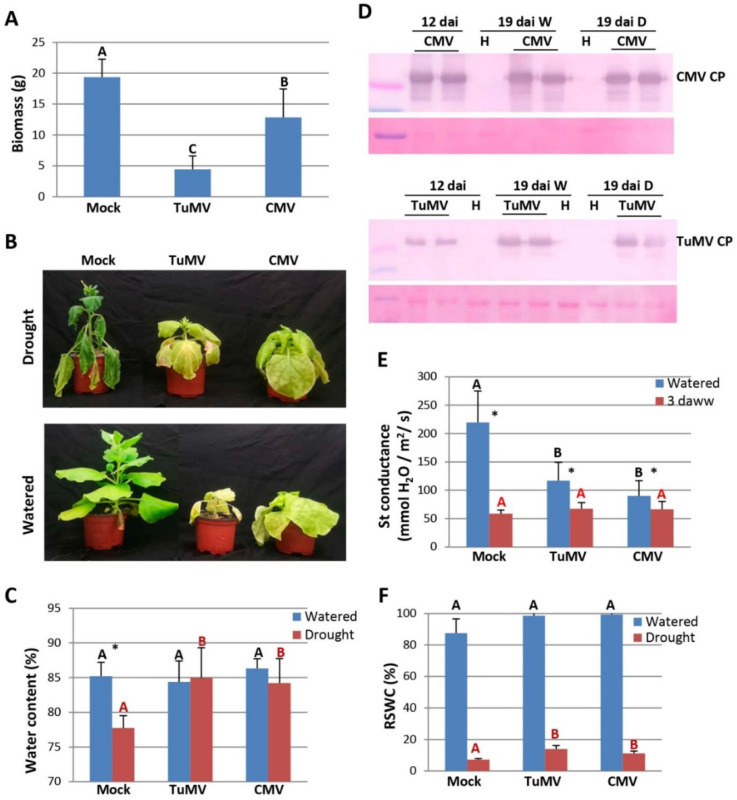
Comparison of tolerance to drought in mock-inoculated, *Cucumber mosaic virus* (CMV)- and *Turnip mosaic virus* (TuMV)-infected *Nicotiana benthamiana* plants. (**A**) Biomass of watered (W) plants at 19 days after inoculation (dai). (**B**) Seven days after the water was withheld (daww) (upper panel), representative plants were photographed together with their watered counterparts (bottom panel). (**C**) Water content percentage in virus-infected and mock-inoculated plants grown under W or drought (D) conditions at 19 dai (7 daww). (**D**) Western blot analysis of protein extracts from plants infected with CMV (upper panels) or TuMV (lower panels) grown under W or D conditions at 12 or 19 dai, using antibodies against CMV CP or TuMV CP. Two independent pooled samples were analyzed for each inoculum. The lower panels show the Ponceau S-stained membrane after blotting, as a control of loading. (**E**) Effect of drought on stomatal conductance in virus-infected and mock-inoculated plants grown under W or D conditions at 3 daww. (**F**) The relative soil water content (RSWC) was measured in each pot of plants grown under W or D conditions. Data represent the means ± standard errors of 18 plants that received the same treatment. Statistical comparisons between means were made among treatments (i.e., Mock, CMV and TuMV) within each watering condition (i.e., watered, drought) by employing Scheffé’s multiple range test (**A**,**E**,**F**) and a Mann–Whitney U test with a Bonferroni correction for multiple comparisons of α to α = 0.016 (**C**). Different letters indicate significant differences (*p* < 0.05). For pairwise comparisons, an asterisk indicates the statistical significance of drought-stressed plants compared to watered plants (*p* < 0.05).

*N. benthamiana* plants were mock-inoculated or inoculated with TuMV and CMV. Virus-infected and mock-inoculated plants were normally irrigated or deprived of watering at 12 days after inoculation (dai) resulting in stress caused by water deficit. The damaging effect caused by viral infection on the biomass of watered plants was higher for TuMV than for CMV at 19 dai (Figure 1A). After withholding water, drought symptoms in mock-inoculated plants first appeared as drooped, curled, or wilted leaves. The prolonged water deficit eventually led to plant collapse and death (Figure 1B). In plants infected with TuMV and CMV, the onset of drought symptoms was delayed by several days and they clearly maintained a reduced wilting appearance compared to mock-inoculated plants throughout the experiment. Water content, stomatal conductance and the relative soil water content (RSWC) are valuable tools for providing information about plant responses to water deficit. The water content of virus-infected and mock-inoculated plants growing under watered and drought conditions was compared at 19 dai, i.e., 7 days after the water was withdrawn (daww). Under drought conditions, average water content was higher in plants infected with TuMV and CMV compared to mock-inoculated plants, indicating that infections by TuMV and CMV promote drought tolerance in *N. benthamiana* (Figure 1C). A similar water content was found in watered plants infected with TuMV and CMV compared to mock-inoculated plants (Figure 1C). Comparative analysis of virus accumulation before and after the water was withdrawn by western blot revealed that the level of CMV CP and TuMV CP in plants grown under drought was similar to that in plants grown under normal irrigation at 19 dai (Figure 1D).

Because a decrease in transpiration rate is an important trait of plant tolerance to drought, measurements of stomatal conductance were calculated in both watered and drought-stressed plants at 15 dai (3 daww) (Figure 1E). Under normal irrigation, infection by CMV and TuMV caused a decrease in conductance compared to mock-inoculated plants. After several days without watering, leaves of mock-inoculated plants showed a sharp drop in stomatal conductance compared to watered plants, whereas leaves of plants infected with either CMV or TuMV exhibited only a smaller reduction in conductance. To determine whether water is similarly depleted in pots of virus-infected and mock-inoculated plants under drought stress, the water content of the soil was measured in both watered and drought-stressed plants at the end of the water deficit period (7 daww; 19 dai). An obvious difference in the RSWC was observed under the two water regimes assayed (Figure 1F). Moreover, a small but statistically significant higher level of RSWC was observed in virus-infected plants compared with mock-inoculated plants under drought stress. Thus, relative differences in soil moisture were correlated with differences in stomatal conductance before the water was withdrawn and tolerance to drought in virus-infected *N. benthamiana* plants. 

### 2.2. CMV-Infected N. Benthamiana Plants Improved Their Reproductive Fitness When Exposed to Drought 

**Figure 2:**
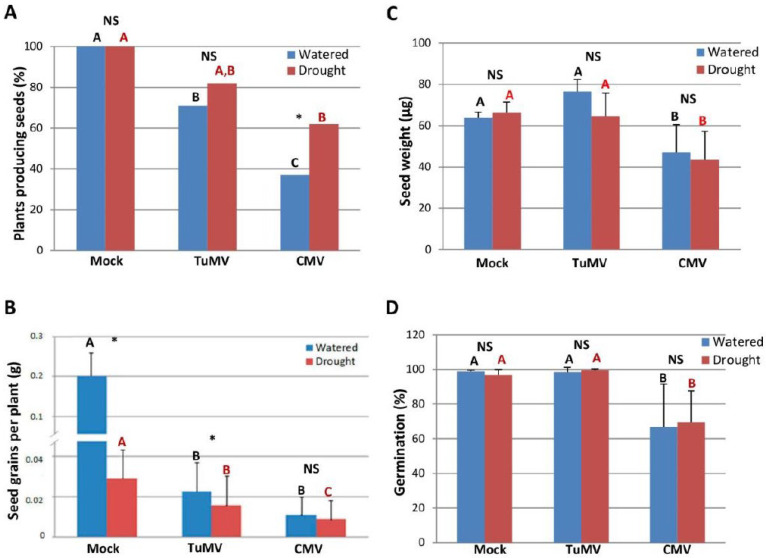
Effect of TuMV and CMV infection on the number of *Nicotiana benthamiana* plants producing seeds, seed grain, seed weight and seed viability. (**A**) Virus-infected and mock-inoculated plants were grown under watered and drought conditions, and the number of plants producing seeds was recorded. (**B**) Seeds were weighted separately after threshing and recorded as seed grain per plant. (**C**) Effect of virus infection on the weight of individual seeds. Seed weight was estimated after determining the weight of 80 seeds derived from each of four to seven plants per treatment. (**D**) Effects of virus infection on seed viability. Seed viability was measured as the germination percentage of approximately 100 seeds per plant, using 3 to 6 individuals per treatment. Statistical comparisons between means were made among treatments within each watering condition by employing Fisher’s exact test with a Bonferroni correction for multiple comparisons of α to α = 0.016 (**A**) and Scheffé’s multiple range test (**B**–**D**). Different letters indicate significant differences. For pairwise comparisons, asterisks indicate significant differences between treatments (Student’s *t*-test, *p* < 0.05); NS: not significative.

The effect of virus infection on the reproductive fitness of both drought-stressed and watered *N. benthamiana* plants was determined by the number of infected plants producing seeds and the seed grains per plant, and by comparing them with those produced by mock-inoculated plants. Under watering conditions, infection by TuMV and CMV conferred a severe detrimental effect on the number of seed-producing plants and on seed grain per plant compared to mock-inoculated plants (Figure 2A,B). Under drought conditions, infection by CMV led to a decrease in the number of plants producing seeds compared to mock-inoculated controls, albeit less severe than under the watered regime. Remarkably, the number of CMV-infected plants grown under drought that produced seeds was statistically higher than that observed in plants infected with CMV under normal irrigation (Figure 2A). By contrast, no statistical differences were observed in the number of TuMV-infected plants producing seeds when grown under watered or drought conditions (Figure 2A). On the other hand, infection by either TuMV or CMV caused a statistical significant reduction on seed grain per plant when compared to mock-inoculated plants, under drought conditions (Figure 2B). However, no significant differences in seed grains were observed between CMV-infected plants that were irrigated or deprived of irrigation (Figure 2B). 

Next, we examined the effect of drought on the weight of individual seeds and seed viability of plants infected with TuMV and CMV compared to mock-inoculated plants (Figure 2C,D). The water regime did not affect the seed weight or rate of germination, regardless if seeds were derived from mock-inoculated or virus-infected plants. Although infection by CMV reduced seed germination by ca. 40% relative to non-infected plants, the viability of seeds and the weight of individual seeds derived from virus-infected plants grown under drought conditions was not differentially affected when compared to those grown under normal irrigation. Altogether, water deficit improved the reproductive fitness of CMV-infected *N. benthamiana* plants, but not that of plants infected with TuMV, when compared to watered, virus-infected plants.

### 2.3. Tolerance to Drought in Virus-Infected Arabidopsis Plants

**Figure 3:**
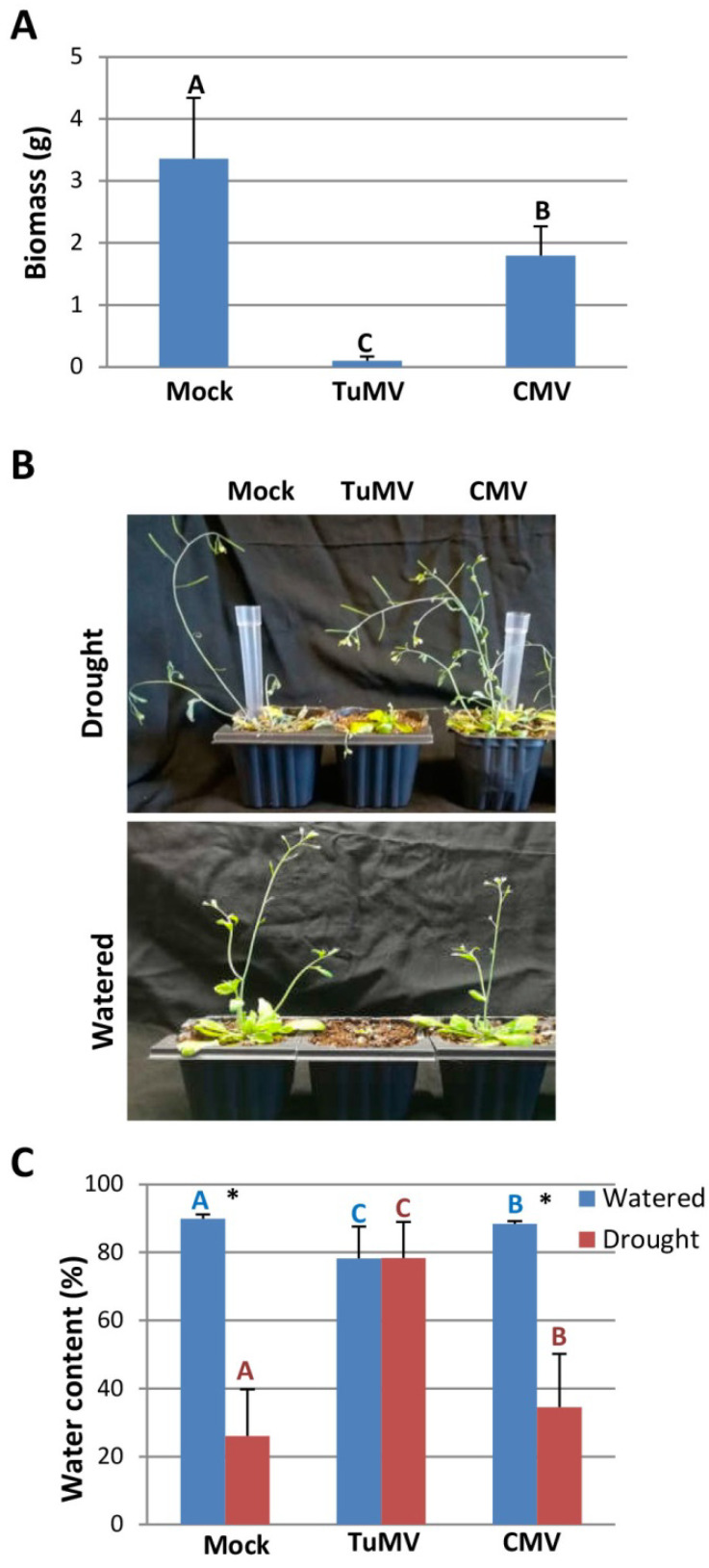
Comparison of tolerance to drought in mock-inoculated, CMV- and TuMV-infected Arabidopsis plants. (**A**) Biomass of watered plants at 30 days after inoculation. (**B**) Twelve daww (upper panel), representative plants were photographed next to their watered counterparts (bottom panel). (**C**) Water content percentage in virus-infected and mock-inoculated plants at 14 daww. Data represent the means ± standard errors of at least 18 plants that received the same treatment. Statistical comparisons between means were made among treatments within each watering condition by employing a Mann–Whitney U test with a Bonferroni correction for multiple comparisons of α to α = 0.016. For pairwise comparisons, asterisks indicate the statistical significance of drought-stressed plants compared to watered plants (Mann–Whitney U test, *p* < 0.05).

Arabidopsis seedlings were either mock-inoculated or inoculated with TuMV or CMV. Virus-infected and mock-inoculated plants were normally watered or subjected to water stress resulting from water withholding at 16 dai. The damaging effect of viral infection on the biomass of watered plants was higher for TuMV than for CMV at 30 dai (Figure 3A). After water deprivation, TuMV- and CMV-infected plants wilted more slowly and displayed milder drought-related symptoms than mock-inoculated plants (Figure 3B). At 30 dai (14 daww), the water content of virus-infected and mock-inoculated plants was compared (Figure 3C). The average water content was higher in TuMV- and CMV-infected plants than in mock-inoculated plants under non-watered growth conditions, indicating that infection by TuMV and CMV promotes tolerance to drought in Arabidopsis. Indeed, the water content in plants infected with TuMV grown under water deficit was similar to that observed in TuMV-infected plants grown under watered conditions.

**Figure 4:**
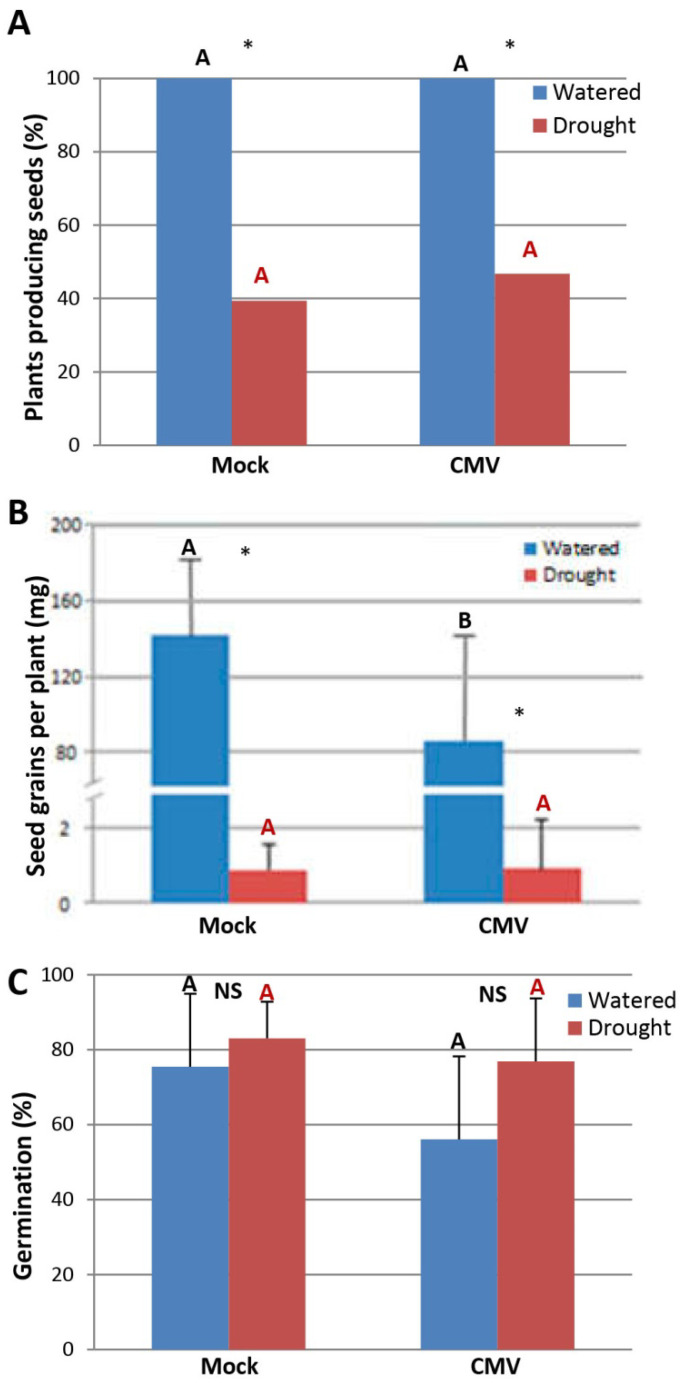
Effect of CMV infection on the number of Arabidopsis plants producing seeds, on seed grain and on seed viability. (**A**) Virus-infected and mock-inoculated plants were grown under watered and drought conditions, and the number of plants producing seeds was recorded. (**B**) Seeds were weighted separately after threshing and recorded as seed grain per plant. (**C**) Effect of CMV infection on seed viability. Seed viability was measured as the germination percentage of approximately 100 seeds per plant, using six individuals per treatment. Statistical comparisons between means were made among treatments within each watering condition by employing Student’s *t*-test (**B**,**C**, *p* < 0.05) and Fisher’s exact test with a Bonferroni correction for multiple comparisons of α to α = 0.025 (**A**). Different letters indicate significant differences. For pairwise comparisons, asterisks indicate significant differences between treatments (Student’s *t*-test, *p* < 0.05); NS: not significative.

In our growth conditions, infection by TuMV in both watered and drought-stressed Arabidopsis plants affected several plant developmental traits, including flower and silique viability, which eventually led to sterility. Thus, the effect of CMV infection on the reproductive fitness of both watered and drought-stressed plants was studied. Infection by CMV did not affect the number of Arabidopsis plants producing seeds compared to mock-inoculated controls, regardless of whether plants were grown under drought or under watered conditions (Figure 4A). By contrast, seed grains per plant were reduced in plants infected with CMV compared to those of mock-inoculated plants when grown under watered conditions. However, negligible differences were observed in seed grains between CMV- and mock-inoculated plants when grown under drought (Figure 4B). Infection by CMV did not affect the viability of Arabidopsis seeds regardless of the water regime (Figure 4C). Thus, infection by CMV did not improve the reproductive fitness of Arabidopsis plants exposed to drought when compared to that of plants infected by CMV grown under watered conditions.

### 2.4. The Effect of Drought on the Transmission of CMV through N. Benthamiana and Arabidopsis Seeds

**Figure 5:**
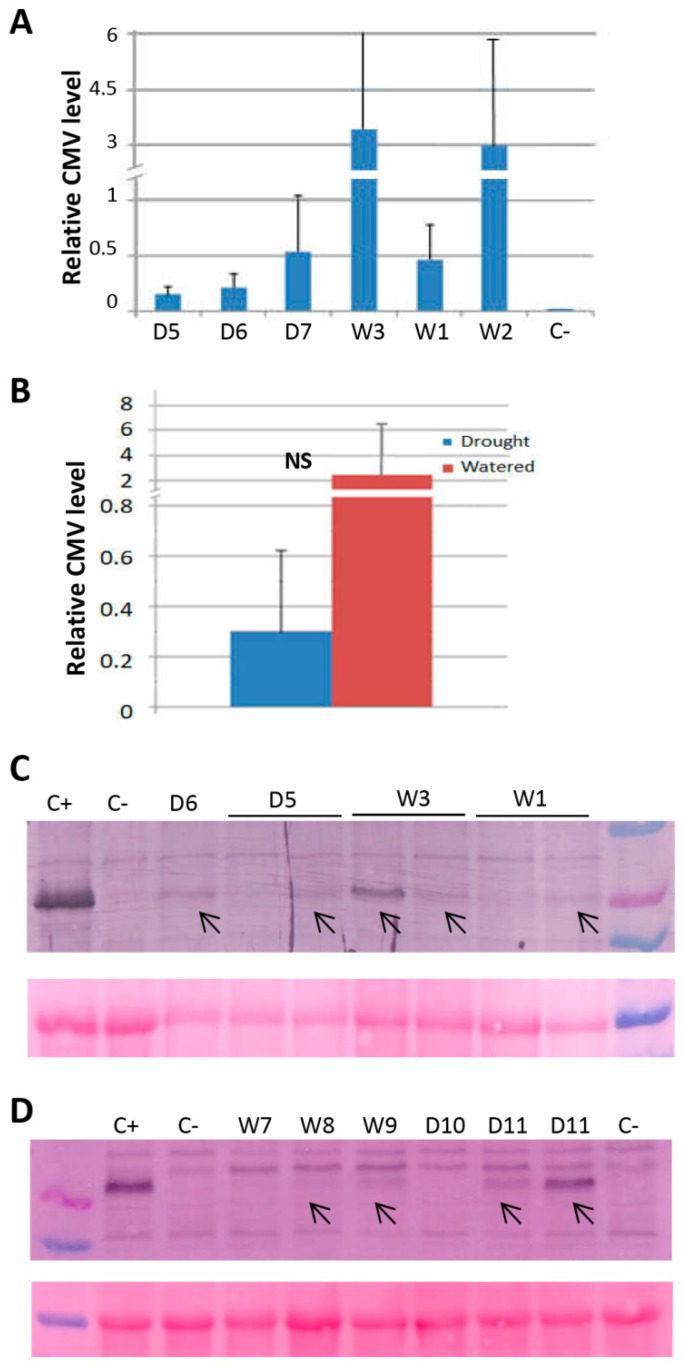
Effect of drought on CMV seed transmission. (**A**) CMV relative levels estimated by RT-qPCR in four pools of *Nicotiana benthamiana* seedlings derived from each of three progenitor plants grown under W and D conditions. (**B**) Average level of CMV estimated by RT-qPCR in progenies derived from watered and drought-stressed *N. benthamiana* plants. NS: not significative (Student’s *t*-test, *p* < 0.05). Western blot analysis of protein extracts derived from pools of *N. benthamiana* (**C**) and Arabidopsis (**D**) seedlings, using antibodies against CMV CP. The lower panel shows the Ponceau S-stained membrane after blotting, as a control of loading. Lane C+ corresponds to an extract from a CMV-infected plant diluted 1:500. Lane C- corresponds to an extract from non-infected seedlings.

To analyze the effect of water deficit on CMV transmission through seeds, progenies derived from infected plants grown under watered or drought treatments were germinated in vitro. Seeds were sterilized to ensure that any positive detection was not the result of virus contamination on the seed coat, but rather the existence of embryonic infection. Four pools of seedlings derived from each of three CMV-infected *N. benthamiana* plants grown under watered and drought conditions were analyzed by real-time quantitative reverse transcription (RT-qPCR), using seedlings derived from non-infected plants as a negative control. CMV was detected in all of the pools of seedlings analyzed, regardless of whether the progenitors were grown under watered or drought conditions (Figure 5A). On average, the relative level of CMV in progenies derived from watered plants tended to be higher than in drought-stressed plants, although there was no statistical significance due to the variability of the samples (Figure 5B). To confirm the above results, pools of seedlings derived from a subset of the progenies analyzed above were assayed by western blot analysis. The CMV CP was detected in most of the progeny pools derived from infected plants grown under watered or drought conditions (Figure 5C, arrows).

We also measured the efficiency of seed transmission of CMV in progenies derived from infected Arabidopsis plants grown under watered or drought conditions. Pools of progenies derived from each of three CMV-infected plants grown under watered and drought conditions were analyzed by western blot. Two out of three progeny pools in each group of plants accumulated CMV CP (Figure 5D, arrows). Altogether, our data showed that water deficit did not significantly affect seed transmission of CMV in *N. benthamiana* and Arabidopsis plants.

After several attempts to detect TuMV in progenies derived from watered and drought-stressed *N. benthamiana* plants, we could not detect seed transmission of TuMV by RT-qPCR and western blot analyses (Figure S1).

## 3. Discussion

Infection by either TuMV or CMV promotes drought tolerance in Arabidopsis and *N. benthamiana* plants, as reported previously for CMV in Arabidopsis [14] and *N. benthamiana* [15], and TuMV in Arabidopsis [32]. In a previous work, infection with *Plum pox virus* (PPV), *Potato virus X* (PVX), a genetically engineered PPV recombinant expressing the PVX RNA silencing suppressor protein P25 (PPV–P25), and mixed PPV/PVX infection supported a positive association between virulence and tolerance to drought [10]. Estimates of virus effects on biomass, reproductive performance and water content in drought-stressed Arabidopsis plants infected with TuMV and CMV shown in this study were also consistent with a positive correlation between virulence and tolerance to drought; that is, infection by TuMV conferred an enhanced drought-tolerant phenotype compared to infection with CMV. However, no such correlation was observed in *N. benthamiana*.

Several works have shown that virus infections influence stomatal development in *Nicotiana* spp. and in Arabidopsis, which was associated with a reduction in transpiration rates [10,33]. In this study, infection by CMV and TuMV caused a decrease in stomatal conductance in *N. benthamiana* under watered conditions, which was correlated with a less pronounced decline in soil water content compared to mock-inoculated plants following withdrawal of water. A reduction of stomatal conductance could facilitate tolerance to drought induced by virus infection, as a reduced transpiration rate is an important survival strategy in the response of plants to drought stress [7]. Plants with decreased stomatal conductance are expected to consume soil water more slowly, avoiding the detrimental consequences of low water potential, and thereby may became more tolerant to water deficit. Nevertheless, other mechanisms not mutually exclusive such as osmotic adjustment, root architecture, responses to abscisic acid (ABA) or effects on salicylic acid (SA)-mediated signaling might also play a role in virus-induced drought tolerance [10,14,15]. In addition, imaging techniques, such as thermal or spectral sensors, could be adopted as high-throughput and non-invasive tools for evaluating drought tolerance in virus-infected plants in breeding programs, when large numbers of genotypes and viruses have to be tested. 

Studies on reproductive performance have previously shown that tolerance to drought in plants infected with viruses was not necessarily associated with an increase in host fitness. For instance, infection caused by the virulent recombinant PPV–P25 was detrimental to reproductive fitness, whereas infection showing moderate virulence (PPV) was able to increase fitness of Arabidopsis plants grown under severe drought conditions [10]. Similarly, the wheat–*Barley yellow dwarf virus* pathosystem shifted from pathogenic to beneficial along gradients of water stress intensity and duration [12]. Nevertheless, caution must be taken when comparing distinct experimental approaches, such as plant species, stress intensity and developmental stage, to assess the effect of virus infection on host fitness under water deficit, as physiological outcomes in different pathosystems are far from unique. Here we employed the experimental approach described by Aguilar et al. [10] for stress imposition. Neither CMV nor TuMV had a positive impact on reproductive fitness following withdrawal of water, as determined by measuring seed grains, seed germination and the number of individuals producing seeds in virus-infected vs. mock-inoculated Arabidopsis plants or *N. benthamiana* plants. By contrast, the damaging effect caused by CMV and TuMV on host reproductive fitness with regard to mock-inoculated plants overcame any beneficial effect associated with virus-induced tolerance to drought in *N. benthamiana*, as previously reported for the virulent recombinant PPV–P25 [10]. In Arabidopsis, seed grains did not decrease following infection with CMV under drought conditions, as it happened when plants were grown under watered conditions. Thus, virus-induced drought tolerance could compensate for decreased production of progeny on Arabidopsis infected with CMV. Nevertheless, an improved reproductive fitness in terms of increased seed grain under drought conditions is not an inevitable outcome of infection in every virus–host pathosystem. An extreme case of detrimental effect on host fitness was that caused by TuMV in Arabidopsis. It has been reported that a striking characteristic of plants infected with the UK 1 strain of TuMV was that most of them failed to develop a flower stalk, and the few flowers were abnormal and mostly nonfertile [34].

Although it could be anticipated that exposures to abiotic stresses would weaken plant defenses and thus make the plants more vulnerable to pathogen infection, there are examples involving different plant–virus combinations where abiotic stresses can modulate plant tolerance toward viruses [20,21,22,23,26]. Here, we experimentally tested the hypothesis that water deficit increases tolerance, estimated as reproductive fitness, of Arabidopsis and *N. benthamiana* plants infected with CMV and TuMV when compared to watered, virus-infected plants. Notably, the number of CMV-infected *N. benthamiana* plants grown under drought that produced seeds increased by 67%, with a 35% increase in total seed grain, compared to plants infected with CMV under normal irrigation, whereas no significant differences were observed upon infection with TuMV. Moreover, no differences in germination frequency were observed in progenies derived from CMV-infected plants when they were grown under watered and drought conditions, albeit CMV reduced seed viability under the two water regimes. A lower seed survival rate has been reported in plants infected with CMV, suggesting that the localization of the virus in the seed reduces its viability [35]. Taken together, these findings highlight a conditional improvement in biological efficacy of *N. benthamiana* plants infected with CMV under water deficit. However, no such conditional phenotype was observed in Arabidopsis plants infected with CMV. From the pathogen side, the increased number of individuals infected with CMV that produce seeds under drought conditions could improve virus fitness through an increased chance of spread in the plant population.

Abiotic stress conditions such as drought have been reported to influence the occurrence and spread of pathogens, and directly affect host–pathogen interactions by altering plant physiology and defense responses [36]. In Arabidopsis, the spread of CaMV infection throughout the host slowed down under water deficit conditions [25]. In this study, a comparative analysis of virus accumulation revealed that the level of CMV in virus-infected plants was similar under the two water regimes, suggesting that the drought-enhanced host tolerance to CMV was not likely due to decreased virus accumulation. In this respect, both CMV and TuMV behaved as tolerant to drought in terms of accumulation in systemic tissues. Plant responses to drought might also have additional side effects on plant tolerance to pathogens. For instance, signaling pathways induced in response to drought may act as a priming stimulus and provide enhanced tolerance against virus infection via activation of interlinked pathways, such as those mediated by reactive oxygen species (ROS) and plant hormones. ABA is a key hormone in plant responses to drought stress [7,17], and, together with SA, it has been shown to promote plant resistance to several viruses, including CMV [37,38,39]. Even more, infection of *N. benthamiana* by CMV induced ABA levels and activated the SA and ABA pathways, thereby disrupting the antagonist interplay between these two signaling pathways [40]. Besides that, deregulation of host metabolism by water deficit increases the generation of ROS, which in turn alter the cellular redox status [41]. Although potentially toxic, ROS would also play a role as secondary signals through the interaction with hypothetical redox-sensing proteins leading to retrograde signaling and changes in the expression of genes associated with virus tolerance. In this sense, increased mitochondrial ROS levels activate the mitochondrial respiratory enzyme alternative oxidase (AOX), and it is known that SA-induced virus resistance is modulated by AOX in Arabidopsis and *N. benthamiana* [42]. Montes et al. have previously shown that tolerance to CMV was associated with increased seed yield, whereas tolerance to TuMV was attained through changes in the plant developmental schedule [43]. It is tempting to speculate that water deficit regulates plant stress responses (hormonal and/or physiological) that would induce resource reallocation from growth to reproduction, and thereby lead to tolerance to CMV. Future work should address how signaling pathways responsible for drought tolerance in plants are interlinked to activate routes and processes responsible for conferring tolerance to viruses.

Several findings derived from this work might have practical implications for crops under a climate change scenario. Infection by CMV improved the reproductive fitness of *N. benthamiana* plants when exposed to drought compared to watered, virus-infected plants. However, virus infection still caused a detrimental effect on host performance when compared to healthy plants even under water deficit. Thus, inoculation with virulent pathogens would not be indicated as a prophylactic measure to control losses in crops caused by viruses under a climate change scenario. However, the isolation of attenuated, or less virulent, viral variants could provide some benefits under drought conditions while preventing the detrimental effects that virulence imposes to plant fitness [44]. Virus seed transmission has a profound impact in plant virus epidemiology. It represents a significant source of primary inoculum that also allows for the long-distance dispersal of the pathogen [45]. Our data indicate that water deficit does not affect the capacity of infected plants to transmit CMV through seeds in the two model hosts used in this study. As total seed grain increased in plants infected with CMV under drought conditions compared to normal irrigation, water deficit would confer a selective advantage for virus dispersal in the plant population via seed transmission and the subsequent transmission by vectors. Although the range of virus–host combinations in this study is limited, our findings would lead to the hypothesis that plants will experience increased tolerance to specific viruses under some of the projected climate change scenarios. This would affect the dynamics between plants and viruses and, eventually, their coevolutionary relationship in both agricultural and natural ecosystems, as the ability to spread through seeds is a major determinant of virus fitness. Furthermore, our study investigated drought-induced alterations in plant–virus interactions in two experimental model hosts. Therefore, further field studies focused on the evaluation of vegetative and reproductive performance should be undertaken in plants with agronomic interest to assess the epidemiological consequences of virus infection under water stress.

## 4. Materials and Methods

### 4.1. Viruses 

The Fny strain of CMV was used as inoculum [46]. A sap extract from CMV-infected tissue was made in phosphate buffered saline (pH 6.8) at 10% (*w/v*), aliquoted and kept at −80 °C, and used as the inoculum source for all mechanical inoculations. The binary vector pCB-TuMV-GFP (GenBank: EF028235.1), which harbors a GFP-tagged cDNA of TuMV UK1, was provided by J. Carrington [47,48]. *Agrobacterium tumefaciens* harboring pCAMBIA1305.1, which contains a gene encoding *β-glucuronidase*, was used as a negative control. 

### 4.2. Plant Materials 

*N. benthamiana* plants were grown in 10 cm-diameter pots filled with a mixture of soil and vermiculite at 3:1 (*v/v*). Four-week-old plants were inoculated with CMV or agro-infiltrated with *A. tumefaciens* bearing TuMV [49]. Plants were grown at 25 °C using a 16 h light photoperiod.

*Arabidopsis thaliana* plants were inoculated in vitro with the indicated viruses as described [50]. At 11 dai, plants were transferred to 5 × 5 cm individual pots filled with a mixture of soil and vermiculite at 3:1 (*v/v*), and covered with a plastic bag to avoid desiccation. Plants were grown at 21 °C using a 16 h light photoperiod. 

### 4.3. Drought, Stomatal Conductance and Water Content Measurements

Plants were bottom-watered for 3 h until saturation of the soil at 12 dai (*N. benthamiana*) or 16 dai (Arabidopsis), and then subjected to complete water withholding (Figure S2). A different set of plants were kept watered over the same period as a control. The position of the trays in the grown chamber was changed twice per week to minimize experimental variation between samples. Analysis of water content in watered and drought-stressed plants was performed at 7 daww according to Xu et al. [15]. Aerial tissues of drought-stressed and watered plants were harvested and fresh weights were measured. Then samples were dried over 7 days at 65 °C. Dry weight was measured and the weight loss for each plant, which is a proxy of the water weight, was calculated. The percentage of water content of each plant was calculated by dividing the water weight by the fresh weight for each sample. The number of *N. benthamiana* plants analyzed in three separate experiments were 19 (Mock), 18 (TuMV) and 14 (CMV) for watered conditions; and 17 (Mock), 18 (TuMV) and 15 (CMV) for drought conditions. For Arabidopsis, the numbers of individuals analyzed in two separate experiments were 18 (Mock), 18 (TuMV) and 20 (CMV) for watered conditions; and 38 (Mock), 37 (TuMV) and 37 (CMV) for drought conditions. 

The RSWC was calculated following the formula: (fresh weight-dry weight)/(initial weight-dry weight) × 100, as described before [51].

Stomatal conductance was measured using a leaf porometer (SC-1 Decagon-T, Decagon Devices, Pullman, WA, USA) at 25 °C and 65% relative humidity. In brief, attached, fully expanded leaves of *N. benthamiana* plants were placed in the chamber and repeat measurements of conductance from 10 plants per treatment were taken on the abaxial side of the leaves.

### 4.4. Reproductive Fitness Assays

The number of *N. benthamiana* and Arabidopsis plants producing seeds and seed grains per plant were recorded at complete senescence (Figure S2). The number of *N. benthamiana* plants analyzed in four separate seed grain experiments were 12 (Mock), 38 (TuMV) and 84 (CMV) for watered conditions; and 12 (Mock), 28 (TuMV) and 58 (CMV) for drought conditions. For Arabidopsis, the number of individuals analyzed in two separate seed grain experiments were 20 (Mock) and 20 (CMV) for watered conditions, and 33 (Mock) and 32 (CMV) for drought conditions. Seeds were weighted individually after threshing and recorded as seed grain per plant. Seed weight was recorded after determining the weight of 80 seeds derived from each of four (Mock and TuMV) or seven (CMV) plants per water regimes. Seed viability was estimated as the germination percentage of approximately 100 seeds per plant. Seeds were washed in a 10% household bleach solution (3.7% active chlorine) for 12 min and washed five times in sterile water. Seeds were then placed in Petri dishes containing Murashige and Skoog medium, stratified for three days at 4 °C, and germinated in a growth chamber at 25 °C, under 16 h light. Germination efficiency was determined after 10 days of cultivation. Fifteen days post-stratification seedlings were pooled in groups of five and tested for the presence of viruses via RT-qPCR and western blot.

### 4.5. Protein Gel Blot Analysis

Viral proteins were analyzed by western blot using specific antisera as described previously [52]. Total proteins were extracted by grounding leaf disks in nitrogen with a pestle, and adding 400 μL of extraction buffer/0.05 g (0.1 M Tris-HCl pH 8, 10 mM EDTA, 0.1 M LiCl, 1% β-mercaptoethanol and 1% SDS). Samples were then boiled and fractionated in 15% SDS-PAGE gels. Gels were wet-blotted onto Hybond-P PVDF membranes (Amersham, GE Healthcare, Buckinghamshire, UK). For the detection of CMV CP, we used a home-made rabbit polyclonal antiserum (1:1000) [52]. For the detection of TuMV CP, a commercial rabbit antibody was used (No. 07049S/500; Loewe Biochemica GmbH, Sauerlach, Germany). Blotted proteins were detected using commercial secondary antibodies (1:5000) and SigmaFast BCIP/NBT substrate tablets (SIGMA Aldrich, Saint Louis, MO, USA). 

### 4.6. Quantitative RT-PCR (qRT-PCR)

For the estimation of viral RNA levels, total RNAs were extracted from five combined seedlings taken at 15 days post-stratification from progenies derived from drought-stressed and watered plants. TRIzol reagent (Invitrogen, Carlsbad, CA, USA) was used to extract total RNA, and DNA contaminants were removed by treatment with a TURBO DNA-free kit (Ambion, Austin, TX, USA). A one-step qRT-PCR was performed using 15 μL of a reaction mix that contained 7.5 μL of Brilliant III Ultra-Fast RT-qPCR Master Mix (Agilent, Santa Clara, CA, USA), 1.8 μL of RNase-free water, 0.75 μL of reverse transcriptase (Agilent), 0.15 μL of 100 mM dithiothreitol (Agilent), 0.3 μM of each primer, and 3 μL of total RNA extract (approximately 10 ng RNA/μL). Primers employed were CMV RNA3-Fw (5’-CTGATCTGGGCGACAAGGA-3´) and CMV RNA3-Rv (5´-GATAACGACAGCAAAACAC-3´) for RNA 3 of CMV; TuMV CP-Fw (5´-GAAGGAGAAGAAGGAGAGAGAGA-3´), TuMV CP-Rv (5´-GTGCAACATCCTTGCCTTTC-3´) for CP of TuMV; and 18S rRNA-Fw (5´-GCCCGTTGCTGCGATGATTC-3´) and 18S rRNA-Rv (5´-GCTGCCTTCCTTGGATGTGG-3´) for normalization. All RT-qPCR assays were performed in a Rotor-Gene Q thermal cycler (Qiagen. Venlo, Limburgh, Netherlands) as described previously [53]. All reactions were performed in triplicate with two replicates of each sample in each run. The relative quantification of PCR products was calculated by the comparative cycle threshold (ΔΔCt) method. 

### 4.7. Statistical Analysis 

Statistical analyses were conducted in the statistical software IBM SPSS Statistics v.20 (IBM Corp). For each analysis, samples were assessed for normality via the Shapiro–Wilk test, and for equality of variances using Levene’s test. For analysis with approximately normally distributed samples of equal variance, one-way ANOVA followed by Scheffé’s post-hoc test was used. Otherwise, a nonparametric Mann–Whitney U test was employed, with the Bonferroni correction for multiple comparisons between samples applied. For comparisons between pairs of means (pairwise comparisons)*,* Student’s *t*-tests or Mann–Whitney U tests were employed, depending on the normality of the data. Fisher’s exact test was employed for differences in proportions.

## Data Availability

The data presented in this study are available within the article.

## Supplementary Materials

The following supporting information can be downloaded at: https://www.mdpi.com/article/10.3390/plants11091240/s1, Figure S1: Attempts to detect *Turnip mosaic virus* (TuMV) in *Nicotiana* *benthamiana*. Figure S2: Schemes illustrating the experimental design.
