# Supplementary material for: Water Deficit Improves Reproductive Fitness in Nicotiana benthamiana Plants Infected by Cucumber mosaic virus
Source: Plants (Basel). 2022 May 4;11(9):1240. doi: 10.3390/plants11091240 (PMC9105947; doi:10.3390/plants11091240)
Supplement: Supplementary file 1 [file plants-11-01240-s001.zip › plants-1670330-supplementary.pdf]

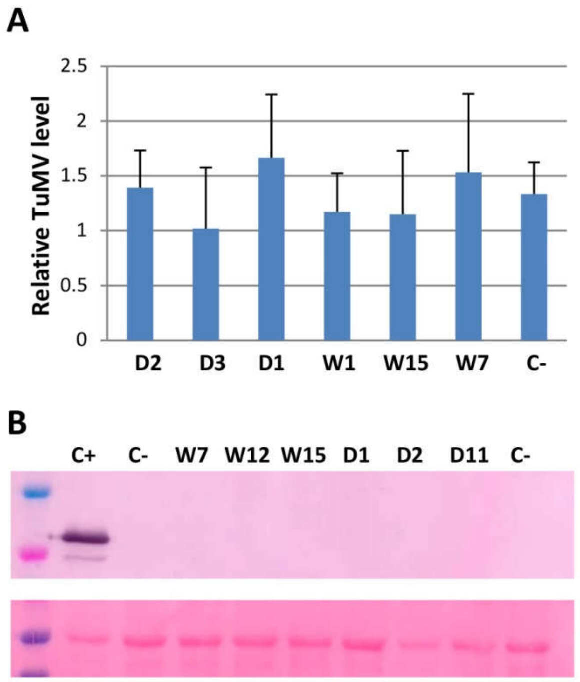

Fig. S1: Attempts to detect *Turnip mosaic virus* (TuMV) in *Nicotiana benthamiana*. (A) RT-qPCR was performed in four pools of seedlings derived from each of three progenitor plants grown under well-watered (W) and drought (D) conditions. (B) Western blot analysis of protein extracts derived from pools of seedlings using antibodies against TuMV CP. The lower panel shows the Ponceau S-stained membrane after blotting, as control of loading. Lane C+ corresponds to extract from a TuMV-infected plant diluted 1:500. Lane C- corresponds to extract from non-infected seedlings.

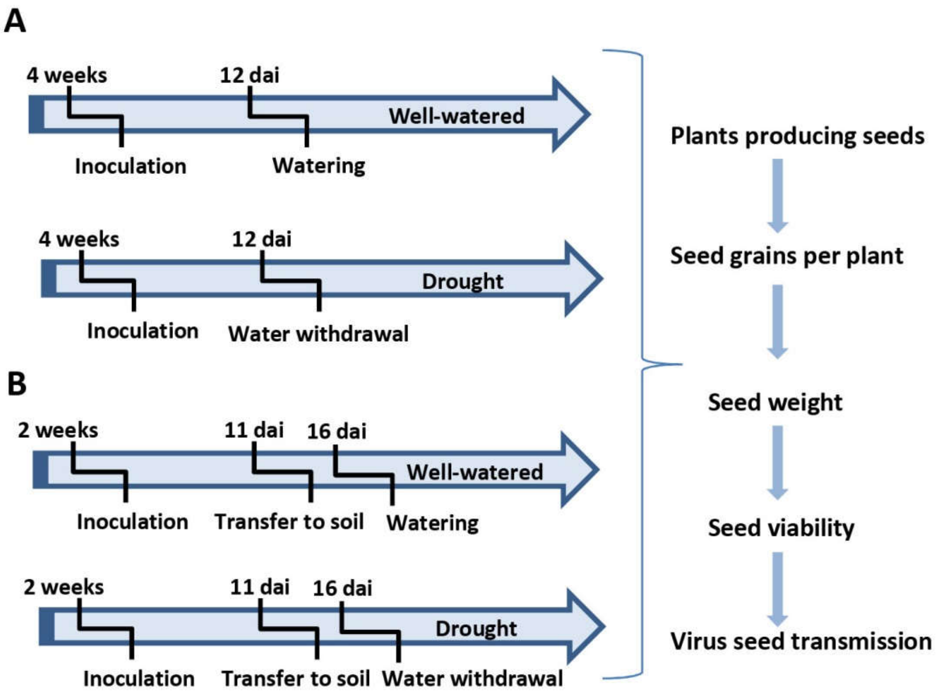

Fig. S2: Schemes illustrating the experimental design for exposing (A) Nicotiana benthamiana and (B) Arabidopsis thaliana plants to well-watered (upper drawings) and drought conditions (lower drawings). The evaluation of reproductive fitness is illustrated. Dai, days after inoculation.
